# Splanchnic vein thrombosis, the onset manifestation in JAK positive Chronic Myeloproliferative Disorders Neoplasms

**Published:** 2011-02-25

**Authors:** AM Vladareanu, V Popov, H Bumbea, M Onisai, A Ilea, C Dobrea, M Miulescu

**Affiliations:** * County Emergency Hospital Pitesti Haematology DepartmentRomania; ** University Emergency Hospital Bucharest Haematology ClinicRomania; *** Ritus Biotec Laboratory Codlea, BrasovRomania; **** Babes Institute Bucharest Morphopatology Department Romania; ***** County Emergency Hospital Pitesti Gastroenterology Department Romania

**Keywords:** portal thrombosis, chronic myeloproliferative disorders, JAK positive

## Abstract

**Background**: Patients with Myeloproliferative Neoplasms–(MPN) have a high risk of thrombotic complications. Portal vein thrombosis is a severe complication, which in many cases, appears at the onset of the disease; the risk factors are related to the presence of qualitatively altered thrombocytes and leucocytes, leading to their activation and appearance of leukocytes–platelet–aggregates; anomalies of portal vein endothelial cells are also implicated. The presence of JAK2V617F mutation increases the risk for splahnic thrombosis.

**Methods and results**: We present three patients with portal vein thrombosis and Budd Chiari syndrome, who were further diagnosed with MPN–the thrombosis was the onset event of the disease

**Conclusion**: Patients were diagnosed with thrombosis of the portal vein before being diagnosed with MPN. Splenectomy was not associated with risk of thrombosis for the two cases in which it was performed; for one case, splenectomy was a therapeutic method to resolve portal hypertension. All patients had homozygous JAK2 mutation, which is associated in recent studies with increased risk of portal, mesenteric thrombosis. The high number of platelet was difficult to control for all patients. Bone marrow biopsy and determination of JAK status are valuable investigations for patients who have splenoportal thrombosis, with no apparent identifiable cause.

## Background

Splanchnic vein thrombosis is a rare complication that can occur in patients with hereditary or acquired thrombophilia, including chronic myeloproliferative syndromes or after splenectomy. Recently, a more frequent association of splanchnic thrombosis with chronic myeloproliferative syndromes (CMPD) JAK positive was reported. The diagnosis of latent forms of CMPD may be difficult when patients have a normal number of cells in the peripheral blood. We present three cases with splanchnic vein thrombosis in which bone marrow biopsy and molecular tests established the diagnosis–JAK positive CMPD

### Case presentation

**Figure 1:**
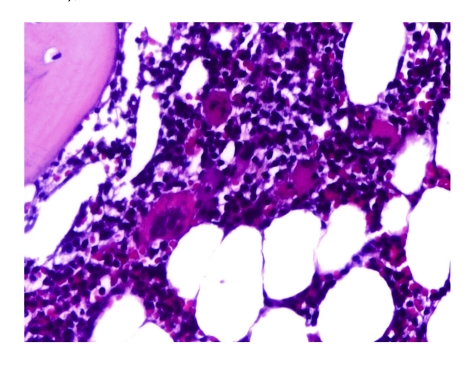
(a and b): Bone marrow trephine biopsy: moderate megakaryocytic hyperplasia with giant hyperlobulated megakaryocytes (H and E stain, 1a–ob 10x; 1b–ob 40x)

**Case 1**–A 53–year–old male with hereditary spherocytosis; the repeated hemolytic events imposed therapeutical splenectomy; between 2004 and 2008 the patient had frequent episodes of hemolysis and severe anemia. After splenectomy, platelet count increased to 2 million/mmc and one month later, he presented extensive thrombosis in the portal territory, superior mesenteric vein and splenic vein, with small abdominal lymph nodes. He received treatment with low molecular weight heparin for 2 months, followed by efficient oral anticoagulation. Platelet count remained over 1 million/mmc. We raised the suspicion of MPN. Clinic examination revealed hepatomegaly (inferior limit 3-4 cm below ribs), without peripheral adenopathy. Laboratory: leukocytosis (WBC 14,600/mmc) with normal differential, thrombocytosis (Platelets 523,000/mmc), Hemoglobin 12. 7g/dl. The peripheral blood smear showed an increased platelet number, platelet aggregates, and giant platelets; the leucocyte and erythrocyte features were normal. Biochemistry showed hepatic cytolysis, low serum iron, increased bilirubin (direct fraction). Serology was negative for HBV, HCV and HIV. We performed a bone marrow trephine biopsy which revealed moderate megakaryocytic hyperplasia–with giant hyperlobulated megakaryocytes, dispersed and in small perivascular groups (Figure 1)

Gomori stain showed a diffuse densification in the reticulin system, with a fine structure (MF=1). 

**Figure 2:**
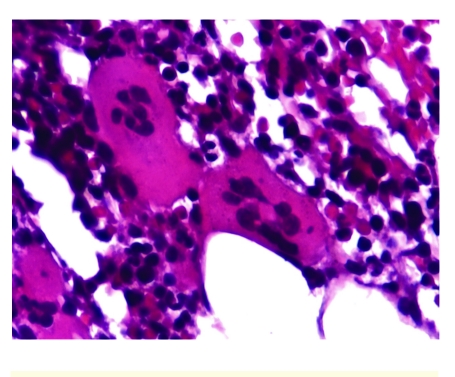
Bone marrow trephine biopsy: diffuse, fine densification of the reticulin network (MF–1) (Gomori stain, ob 10x)

Serum erythropoietin was normal–20U/ml. We performed testing for JAK2V617F mutation–homozygote status was present. The diagnosis was: unclassifiable chronic myeloproliferative neoplasm, JAK positive homozygous, associated with hereditary spherocytosis and portal hypertension (due to portal vein thrombosis). To assess the severity of portal hypertension and to highlight other areas of extramedullary hematopoiesis we performed upper abdominal endoscopy, which revealed severe esophageal mycosis, without lesions on the stomach. Abdominal ultrasound scan–right lobe of liver moderately increased–185 mm, with normal structure, presence of portal hypertension (12 mm the caliber of splenoportal vein). CT scan revealed small lymph nodes above and below the diaphragm. The patient received treatment with Hydrea 1gr/day associated with oral anticoagulant (Sintrom) according to INR value. We also took into consideration Anagrelid as a therapy option–it will be initiated soon. Interferon was excluded because the patient is depressive. Platelet count was maintained between 5–700,000/mmc.

**Case 2**: A 29–year–old male with a history of hematemesis in the last 7 years, due to grade IV esophageal varices (endoscopy); abdominal CT scan: extended thrombosis of splenoportal axis. The splenectomy was performed, associated with shunts for decreasing portal hypertension. Three months after splenectomy, platelet count was over 800,000/mmc, the peripheral blood smear showed increased number of platelet with megathrombocytes and giant form, fragmented of megakaryocytes, large clumps of platelets. We raised the suspicion of MPN. Bone marrow trephine biopsy established diagnosis of ET and PCR exam–V617F mutation on JAK2 gene, homozygous pattern. The patient received treatment with Roferon 6 mill. daily. The platelet count maintained around 600,000–700,000/mmc. 

**Figure 3:**
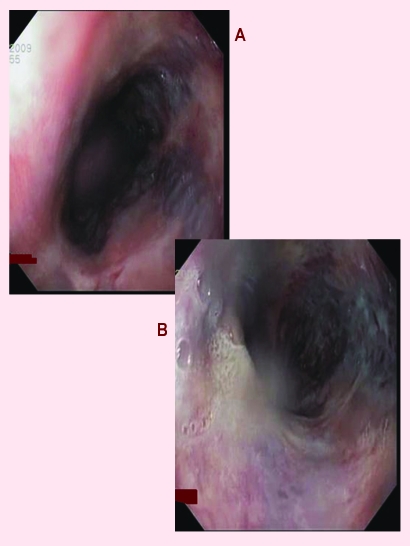
Upper digestive endoscopy–patient with JAK positive MPN–esophageal varices 2nd degree.

**Case 3**. A 54–year–old male, smoker, chronic alcohol consumer was referred to our department for rapid volume increase of the abdomen and diffuse abdominal pain. Objective exam: facial plethora, conjunctival hyperemia, jaundice, cyanosis of the nails, hepatosplenomegaly, ascitis. Laboratory screening showed pancytosis with erytrocytosis predominance (Hb 20g/dl, Ht 62,6%, WBC 18000/mmc with neutrophilia, 1% Eos, 1% Baso; PLT 1000000/mmc), hepatocytolysis (AST, ALT–15xN), cholestasis (GGT= 3xVN, ALKP= 2xVN, BR= 3xVN), all coagulation times spontaneously prolonged, hypoalbuminemia. Bone marrow biopsy: hyperplasia on all lineages, highly suggestive pattern for chronic myeloproliferative disorder, without medullar fibrosis. Endogen erythropoietin–decreased (3mUI/ml). PCR exam –V617F mutation on JAK2 gene, homozygous pattern. Hematological diagnosis was Polycythemia Vera.

**Figure 4:**
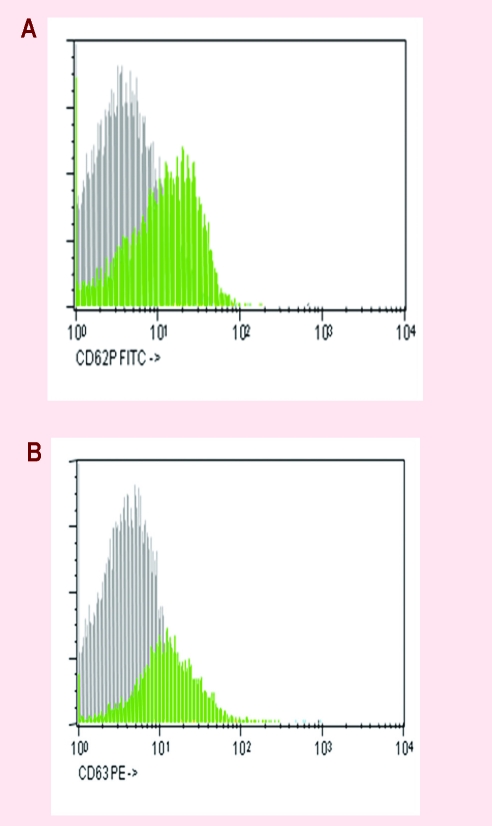
The high expression of activation markers of platelets–CD62P and CD 63.

For all three patients, we assessed the expression of several platelet markers by flowcytometry: CD61/CD41–fibrinogen receptor, corresponding to GPIIb–IIIa–aggregation receptor, CD42a/CD42b–von Willebrand receptor, corresponding to GPIb–IX–adhesion receptor, CD62P–P–selectin–membranary activation receptor, CD63–granulophysin –activation receptor. The results showed a state of platelet activation with increased expressions of CD62P and CD63. (Figure 4) 

We also obtained a significant reduced expression of adhesion markers (CD42b more than the CD42a) for all patients, low expression of CD41 without any differences in CD61 expression.

Platelet function was tested by platelet aggregation studies. We obtained normal response for ADP, collagen and epinephrine for these patients, although other patients with MPN had low response especially for epinephrine. The response for ristocetin was low for one of the patients. 

**Figure 5:**
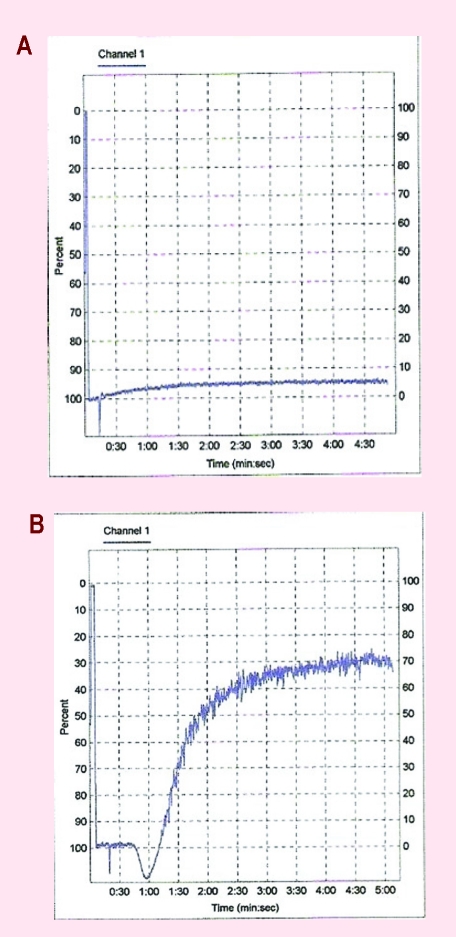
The low platelet aggregation for ristocetin and normal platelet aggregation for collagen in the CMPD JAK2 positive patient.

**Figure 6:**
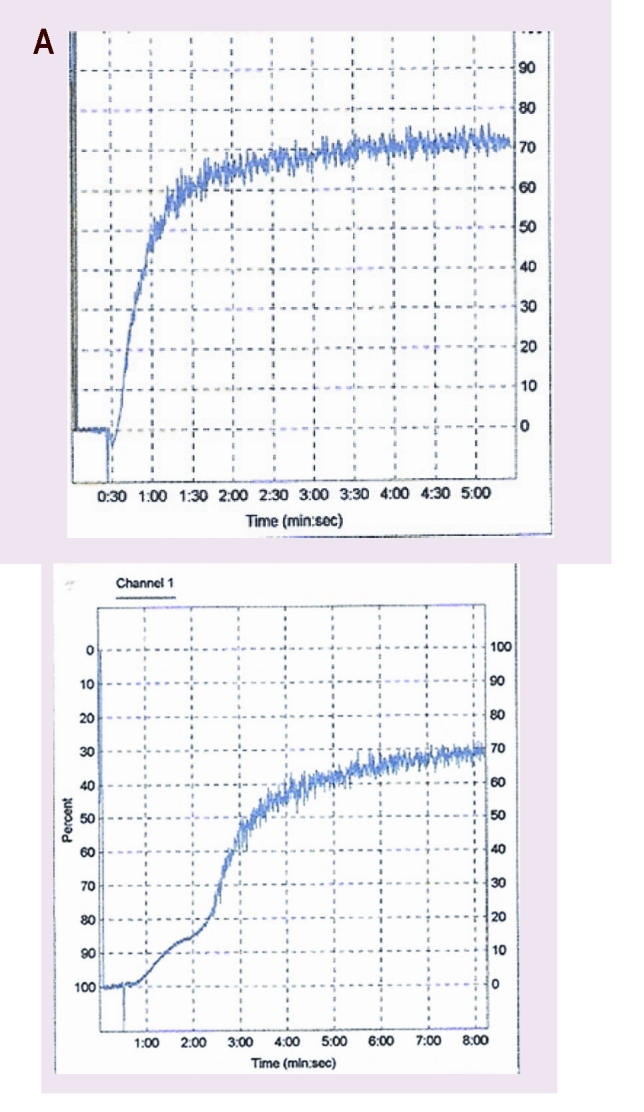
Normal platelet aggregation for ADP and epinephrin in the CMPD JAK2 positive patient

**Discussion**: Portal vein thrombosis or Budd Chiari Syndrome is a rare disorder; chronic myeloproliferative disorders neoplasms (MPN) represent the most common cause. Baxter et al identified the association of JAK2 mutation in 59% of patients with Budd–Chiari syndrome (BCS); Smalberg et al [Hematologica 2006 [1]] found a 41% prevalence of this mutation in BCS patients, on a group of 40 patients with primary non–malignant BCS. Thus, it is necessary to establish the importance of mutation detection in identifying the occult myeloproliferative syndromes [2]. In some cases, the presence of normal or slightly elevated leukocyte or platelet count can complicate the diagnosis. In the first two cases, the diagnosis was made after splenectomy, highlighted by the presence of an abnormal haematological picture, when a high number of platelets persisted for a long time and raised the suspicion of coexistence with MPN. Recent studies which included JAK2 positive patients, showed the presence of morphological and functional changes of endothelial cells corresponding port system. Circulating endothelial progenitor cells and liver endothelial cells may harbour the JAK2 mutation in patients with chronic myeloproliferative disorders, especially in patients who associate Budd Chiari syndrome, demonstrating the role of these cells in the pathogenesis of thrombosis, which may complicate the evolution of MPN. [3] The interaction between endothelial cells, white cells and platelets is achieved by complex mechanisms involving many receptors. These receptors may reveal the status of activated platelets and leukocytes (neutrophils and monocytes). They are in high number on the surface of platelet or leukocyte membrane and may explain the increased interfacing between endothelium and platelet or leukocyte. These receptors are CD11b, CD14, CD62P, CD63. P–selectin expression–basal or after stimulation–is increased in patients with MPN comparative positive JAK2 wild type allele, which shows the role of JAK2 in the modulation of activated status of platelets. P selectin has an important role in activating and selecting leukocyte at the site of endothelial lesion. In addition, JAK2 mutation is involved in activating the leukocyte and the coagulation cascade, in endothelial injury, in producing of leukocyte–platelet aggregates.[3] The presence of leukocyte–platelet aggregates and microparticles (endothelial cell platelet aggregates) in blood circulation is more common in patients with ET and PV. The most common are CD11b/CD62P and CD11b/CD42b aggregates. These aggregates decrease in patients with MPN treated with Aspirin. The most sensitive method of detection is flow cytometry [3,4,5]. These explain thrombophilia and increased risk of thrombosis in patients with chronic myeloproliferative disorders, particularly those with JAK2 mutation present. Increased risk of thrombosis in patients with MPN is due to resistance to activated C protein, which correlates with homozygous JAK2 status, with protrombotic role.[6] Monocytes from JAK2 positive patients with PV and especially ET have an increased capacity for synthesis of tissue factor. Increased level of tissue factor, associated with low levels of S protein, II factor, V factor and inhibitor of tissue factor, have been observed in patients with JAK2 positive MPN, explaining the tendency to thrombosis in these patients.[3] Also, leukocytosis and increased percentage of activated basophils have important role in thrombosis. [3,6] 

In patients considered for this study, an increased level of CD62P expression and CD 63 was observed, corresponding activated status of platelets. The expression of CD41 receptors was low and it was correlated with low platelet aggregation for ristocetin in one patient with JAK positive MPN. The expression of CD42a and CD 42b is low but platelet aggregation to collagen, ADP and epinephrine was normal, which shows changes both quantitative and especially qualitative of platelet receptor GPIIbIIIa. The platelet aggregation for ADP, collagen and epinephrine was more reduced in patients with MPN than controls, especially for epinephrine. 

### Conclusions

Patients with MPN JAK2 positive have a high incidence of splanchnic thrombosis. Determination of JAK2 mutation is useful for investigating the aetiology of portal vein thrombosis or Budd Chiari syndrome, if the aetiology of thrombosis is not obvious. This investigation may be followed by bone marrow biopsy in some cases heralded by haematological abnormalities. 

### Acknowledgements

Acknowledgements: The current study is part of PhD thesis of Dr. Viola Popov, and it was performed with the collaboration of the Haematology Department, Emergency University Hospital Bucharest, special thanks to Diana Cisleanu, MD; Irina Voican, MD; Mihaela Gaman, MD. The authors declare no conflict of interests.
